# Supplementary material for: Kinetochore dynein is sufficient to biorient chromosomes and remodel the outer kinetochore
Source: Nat Commun. 2024 Oct 21;15:9085. doi: 10.1038/s41467-024-52964-5 (PMC11494143; doi:10.1038/s41467-024-52964-5)
Supplement: Supplementary file 1 — Supplementary Information [file 41467_2024_52964_MOESM1_ESM.pdf]

Supplementary Information

for

Kinetochore dynein is sufficient to biorient chromosomes and remodel the outer kinetochore

Bram Prevo[1,2], Dhanya K. Cheerambathur[1], William C. Earnshaw[1], Arshad Desai[2,3,4]

[1]Wellcome Centre for Cell Biology, University of Edinburgh, Max Born Crescent, Edinburgh EH9 3BF, Scotland, UK

[2]Ludwig Institute for Cancer Research, La Jolla, California 92093, USA

[3]Department of Cell and Developmental Biology, School of Biological Sciences, University of California, San Diego, La Jolla, CA 92093, USA

[4]Department of Cellular and Molecular Medicine, University of California San Diego, La Jolla, California 92093, USA

@: Corresponding authors

Email: bram.prevo@ed.ac.uk, abdesai@ucsd.edu

Phone:(858)-534-9698

Address: CMM-E Rm 3052, 9500 Gilman Dr, La Jolla, CA 92093-0653

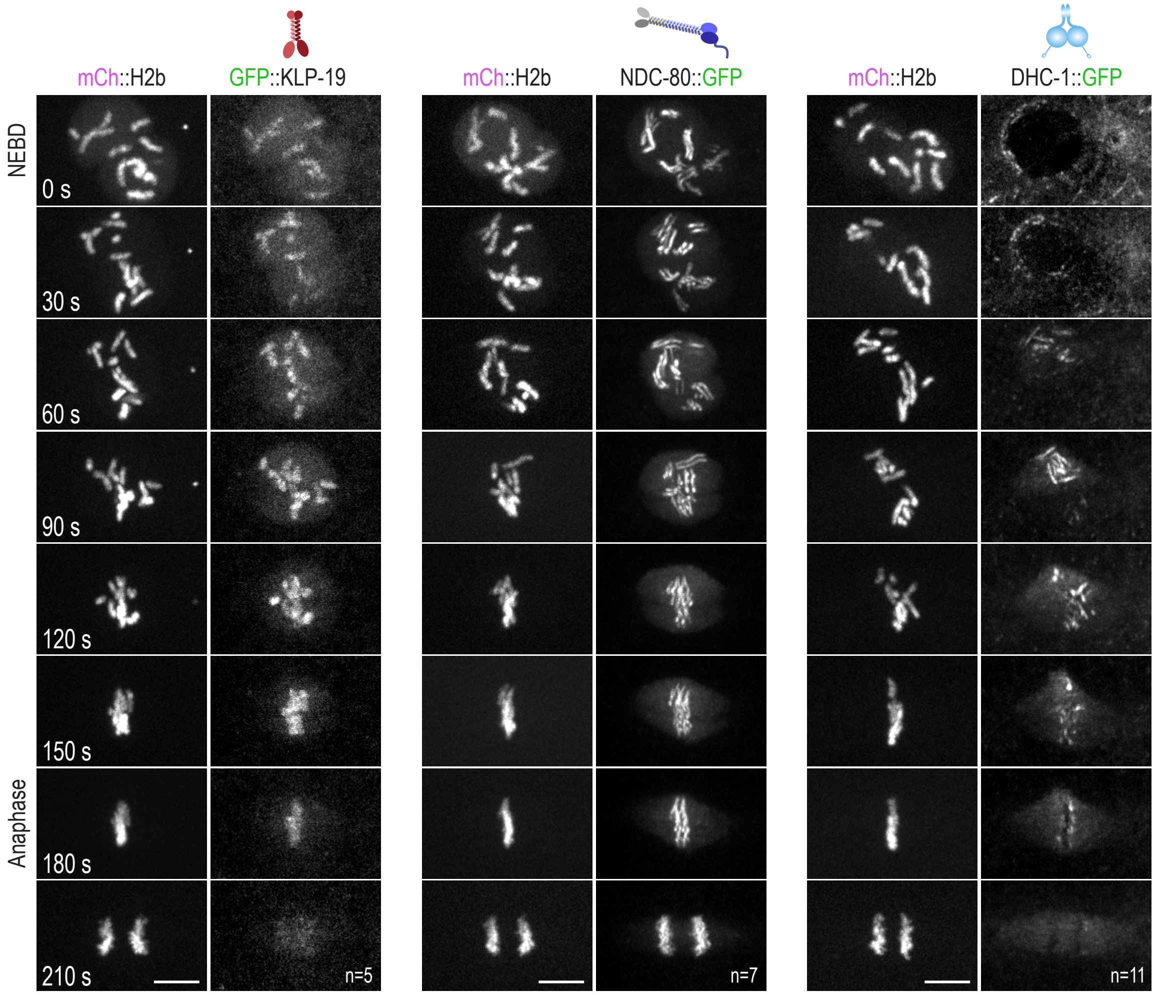
mCh::H2b
GFP::KLP-19
mCh::H2b
NDC-80::GFP
mCh::H2b
DHC-1::GFP
NEBD
Anaphase
0 s
30 s
60 s
90 s
120 s
150 s
180 s
210 s
n=5
n=7
n=11

Supplementary Figure 1. Localization of the three major microtubule-targeting factors during mitosis in the one-cell *C. elegans* embryo.

Image panels from timelapse sequences of *in situ*-tagged GFP fusions of the chromokinesin KLP-19, the NDC-80 subunit of the Ndc80 complex, and the dynein heavy chain DHC-1. mCherry::H2b was used to visualize chromosomes. Numbers on lower left of the first column of mCherry::H2b images indicates time in seconds relative to NEBD. Scale bars, 5 µm. *n* is the number of embryos analyzed.

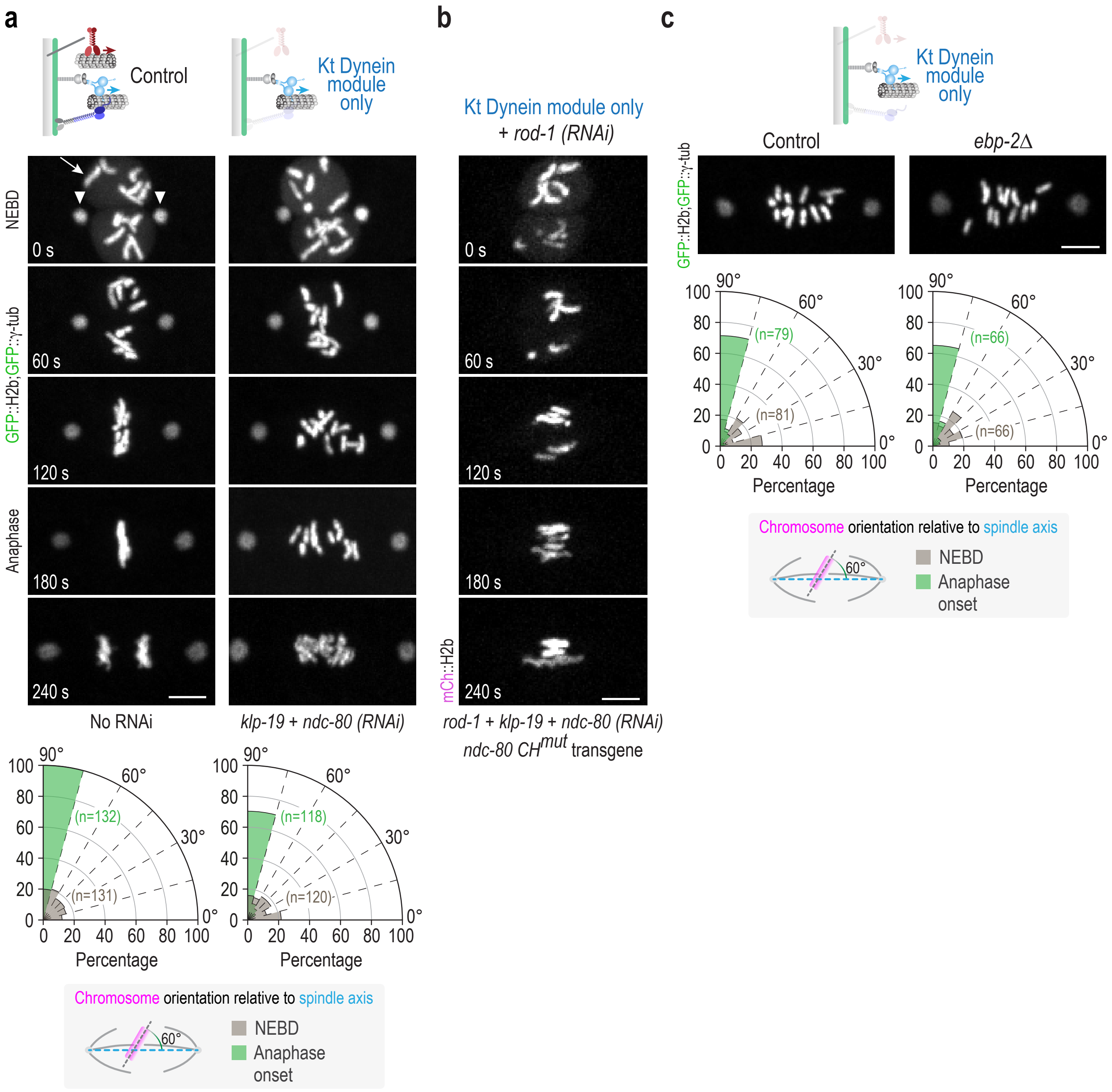
a
Control
Kt Dynein module only
NEBD
GFP::H2b;GFP::γ-tub
Anaphase
0 s
60 s
120 s
180 s
240 s
No RNAi
klp-19 + ndc-80 (RNAi)
90°
60°
30°
0°
100
80
60
40
20
0
(n=132)
(n=131)
(n=118)
(n=120)
0 20 40 60 80 100
Percentage
Chromosome orientation relative to spindle axis
60°
NEBD
Anaphase onset
b
Kt Dynein module only + rod-1 (RNAi)
mCh::H2b
rod-1 + klp-19 + ndc-80 (RNAi)
ndc-80 CH^mut transgene
c
Kt Dynein module only
Control
ebp-2Δ
(n=79)
(n=81)
(n=66)
(n=66)

Supplementary Figure 2. Additional analysis of the kinetochore dynein module-only state.

a Comparison of chromosome behavior in control embryos and embryos co-depleted of KLP-19 and NDC-80 to create the kinetochore dynein module-only state. In contrast to the condition shown in *Fig. 2a*, no transgene encoding the microtubule binding-defective NDC-80 CH^{mut} was present. Graphs below plot chromosome angle relative to the spindle axis, measured as in *Fig. 1e*, at NEBD and anaphase onset. *n* represents the number of chromosomes measured. Scale bar, 5 µm. **b** Representative images from a timelapse series following removal of ROD-1 in the condition used to generate the kinetochore dynein module-only state. Text below the panel indicates the specific perturbations used in this experiment. The 180 s panel from this timelapse sequence is shown in *Fig. 3a*; quantification of chromosome angles relative to the spindle axis for this condition is also shown in *Fig. 3a*. Scale bar, 5 µm. **c** Analysis of the kinetochore dynein module-only state in control and *ebp-2*Δ embryos. Loss of EBP-2, which prevents plus end tracking of dynein, did not significantly affect chromosome orientation, consistent with RZZ-SPDL-1-recruited dynein being responsible for the orientation function. Scale bar, 5 µm. Source data are provided as a Source Data file.

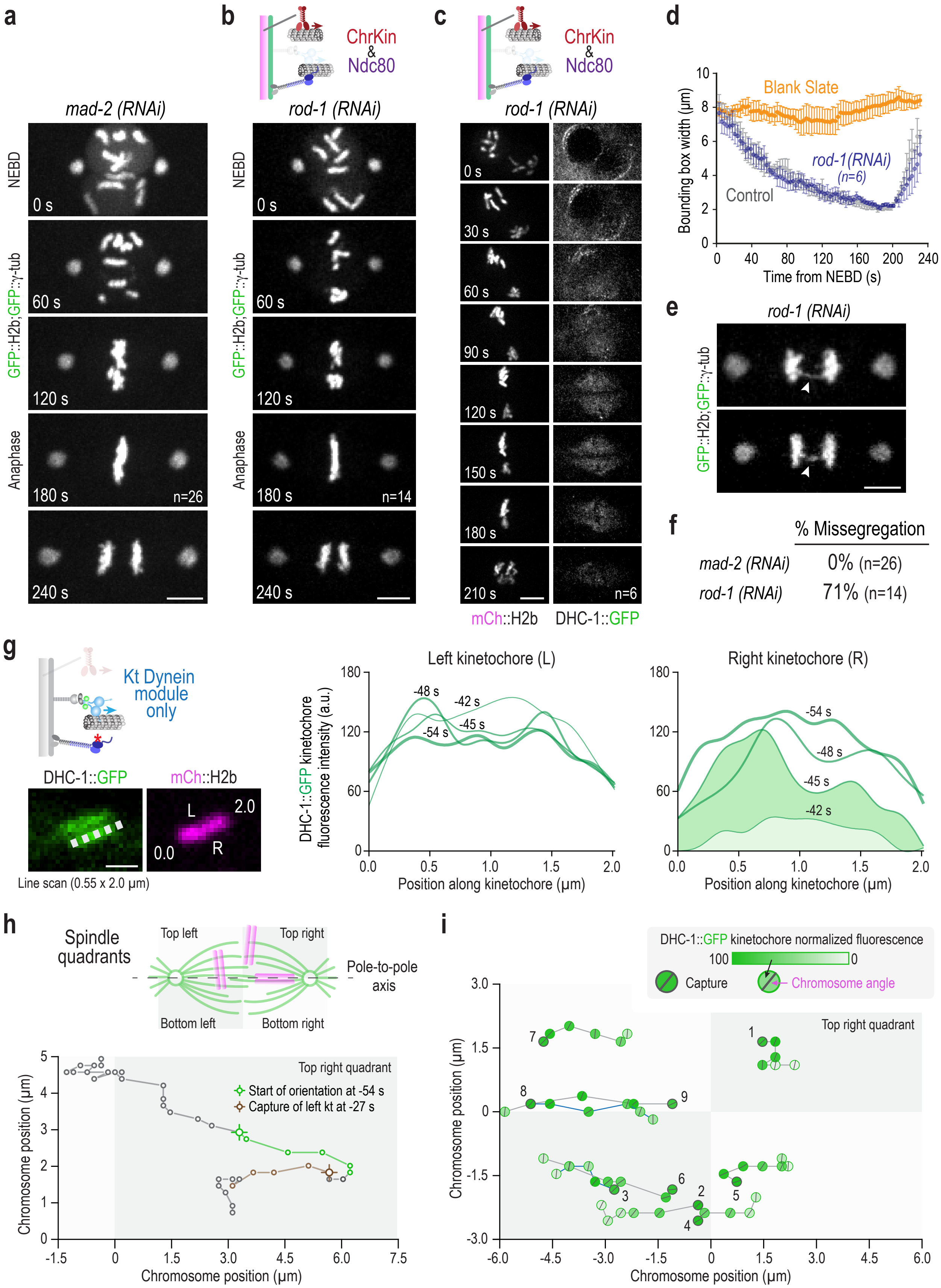
a
b
c
d
ChrKin
&
Ndc80
mad-2 (RNAi)
rod-1 (RNAi)
NEBD
Anaphase
GFP::H2b;GFP::γ-tub
0 s
60 s
120 s
180 s
240 s
n=26
n=14
30 s
90 s
150 s
210 s
n=6
mCh::H2b
DHC-1::GFP
Blank Slate
rod-1(RNAi)
(n=6)
Control
Bounding box width (µm)
Time from NEBD (s)
e
f
% Missegregation
mad-2 (RNAi) 0% (n=26)
rod-1 (RNAi) 71% (n=14)
g
Kt Dynein module only
L
R
2.0
0.0
Line scan (0.55 x 2.0 µm)
Left kinetochore (L)
Right kinetochore (R)
DHC-1::GFP kinetochore fluorescence intensity (a.u.)
Position along kinetochore (µm)
-48 s
-42 s
-54 s
-45 s
h
Spindle quadrants
Top left
Top right
Bottom left
Bottom right
Pole-to-pole axis
Top right quadrant
Start of orientation at -54 s
Capture of left kt at -27 s
Chromosome position (µm)
i
DHC-1::GFP kinetochore normalized fluorescence
100
0
Capture
Chromosome angle

Supplementary Figure 3. Analysis of checkpoint inhibition and of the chromokinesin–Ndc80 module combination created by removal of kinetochore dynein, and of kinetochore dynein removal and chromosome positioning during dynein-mediated orientation.

a Chromosome dynamics in embryos depleted of the spindle checkpoint protein MAD-2. Loss of MAD-2 does not have any significant effect on chromosome segregation (*see also panel e*). Scale bar, 5 µm. **b, c** Chromosome dynamics (*b*) and dynein heavy chain (DHC-1) localization (*c*) in embryos lacking the kinetochore dynein module, where chromokinesin and the Ndc80 module are both present. Scale bars, 5 µm. **d** Quantification of chromosome dispersion on the spindle performed as in *Fig. 1d*. The Control and Blank Slate curves are the same as in *Fig. 1e* and are plotted to aid comparison. Error bars are the 95% confidence interval of the mean (CIM). *n* is number of embryos analyzed. **e** Examples of anaphase segregation defects observed in the absence of the kinetochore dynein module. Scale bar, 5 µm. **f** Summary of missegregation events observed in anaphase of one-cell embryos. *n* is number of embryos imaged. These data indicate that inhibition of the spindle checkpoint does not account for the segregation defect observed in the absence of the kinetochore dynein module. **g** Kinetochore-dynein distribution on individual sister kinetochores quantified using a line scan (dashed grey line) while the chromosome is orienting (data corresponding to the panels displayed in the boxed region of *Fig. 4b*). The right kinetochore shows asymmetric loss of kinetochore dynein along its length during chromosome orientation. To highlight this asymmetry, the -45 s and -42 s curves are shaded. Scale bar, 1.5 µm. **h** Position on the spindle of the chromosome depicted in the time series in *Fig. 4b*. The green symbols correspond to the green highlighted region in *Fig. 4b*. **i** Chromosome positions on the spindle corresponding to the analysis of kinetochore dynein fluorescence intensity and chromosome angles for the 9 kinetochores in *Fig. 4c*. Asymmetric loss of dynein, similar to what is shown in panel g, was observed for all 9 chromosomes. Source data are provided as a Source Data file.

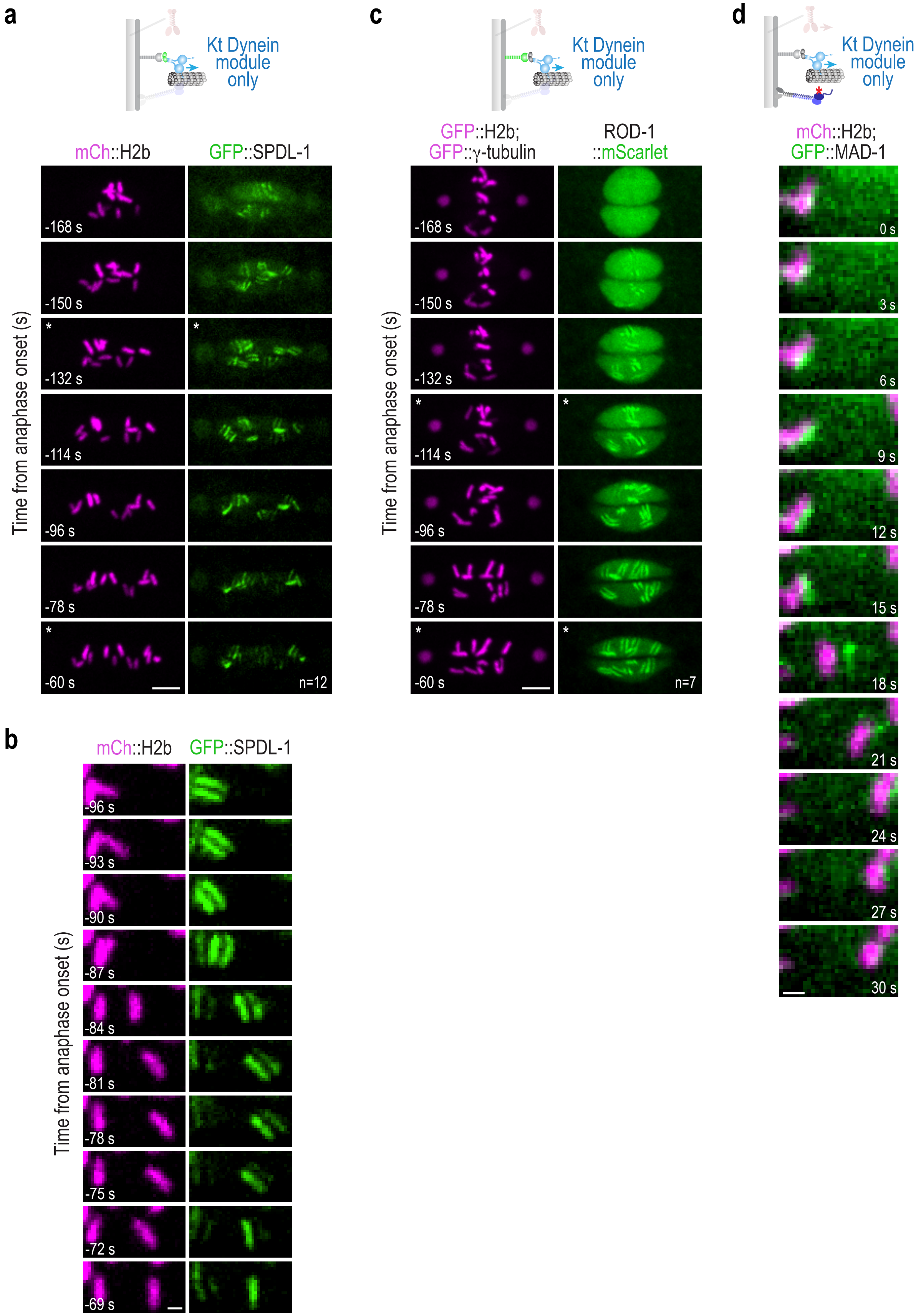

a
Kt Dynein module only
mCh::H2b
GFP::SPDL-1
Time from anaphase onset (s)
-168 s
-150 s
-132 s
-114 s
-96 s
-78 s
-60 s
n=12
b
mCh::H2b
GFP::SPDL-1
Time from anaphase onset (s)
-96 s
-93 s
-90 s
-87 s
-84 s
-81 s
-78 s
-75 s
-72 s
-69 s
c
Kt Dynein module only
GFP::H2b; GFP::γ-tubulin
ROD-1 ::mScarlet
Time from anaphase onset (s)
-168 s
-150 s
-132 s
-114 s
-96 s
-78 s
-60 s
n=7
d
Kt Dynein module only
mCh::H2b; GFP::MAD-1
0 s
3 s
6 s
9 s
12 s
15 s
18 s
21 s
24 s
27 s
30 s

Supplementary Figure 4. Imaging of GFP::SPDL-1, ROD-1::mScarlet and GFP::MAD-1 in the kinetochore dynein module-only state.

a, b Images of *in situ*-tagged GFP::SPDL-1 and **c** ROD-1::mScarlet in the kinetochore dynein module-only state. *(a)* & *(c)* show whole spindle views; *(b)* shows SPDL-1 dynamics on a single orienting chromosome. Note that the transgene encoding NDC-80 CH^{mut} was not present. SPDL-1 behaved similarly to DHC-1, in that kinetochore-autonomous removal was observed. However, ROD-1 behaved distinctly–its levels were maintained at oriented kinetochores. Scale bars, 5 µm in *(a)* & *(c)* and 1 µm in *(b)*. *n* is the number of embryos analyzed. * marked panels from *(a)* and *(c)* are shown in *Fig. 5A*. **d** Image sequence of GFP::MAD-1 in the kinetochore dynein module-only state. The high signal of GFP::MAD-1 in the spindle region, together with its later recruitment relative to DHC-1, made imaging its dynamics on single kinetochores challenging. Nonetheless, chromosomes with kinetochore MAD-1 signal exhibited orientation-coupled removal from kinetochores. In (*d*), the first frame was arbitrarily set to 0 s. Scale bar, 1 µm.

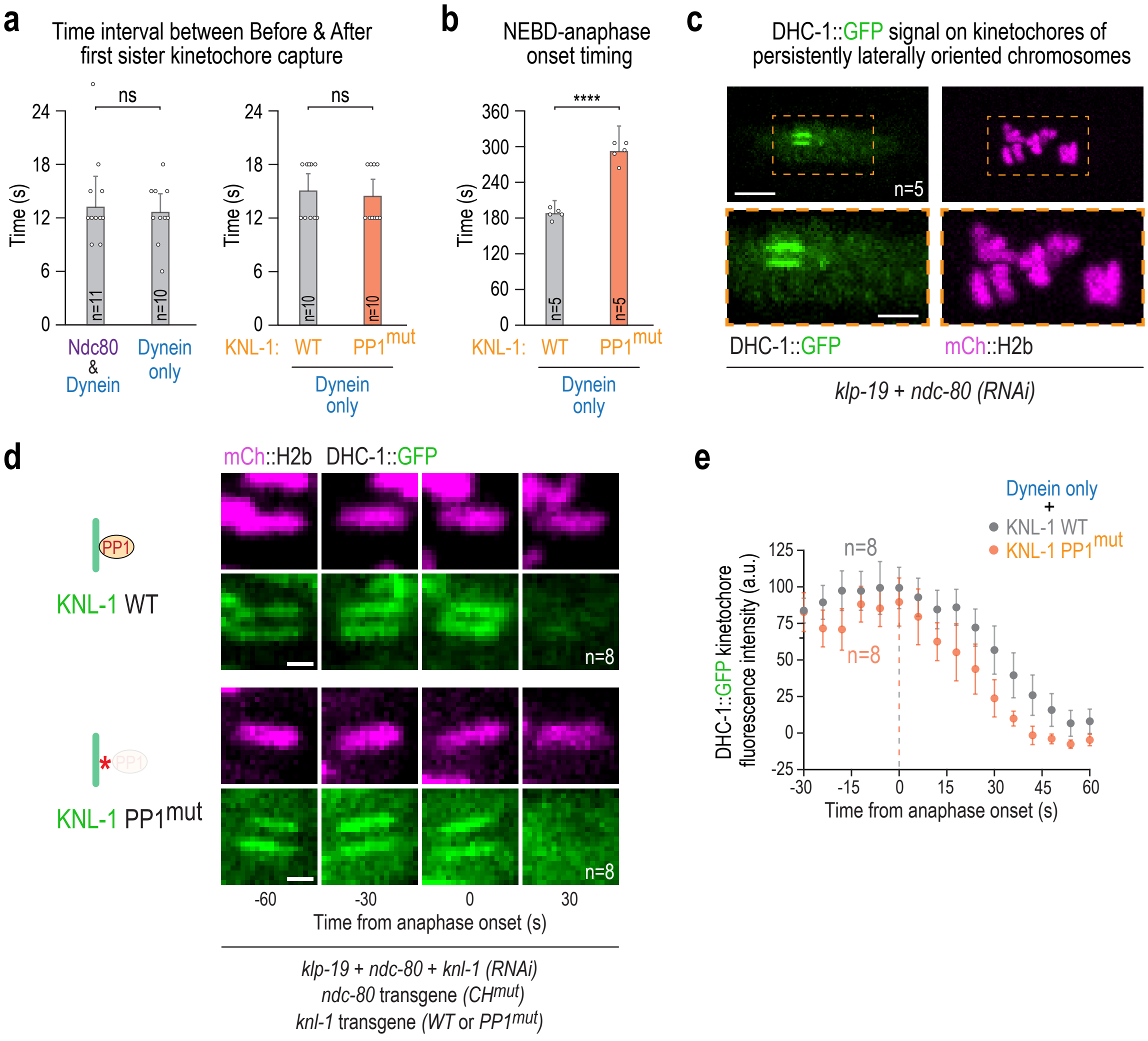

a
Time interval between Before & After first sister kinetochore capture
Time (s)
ns
n=11
n=10
Ndc80 & Dynein
Dynein only
KNL-1:
WT
PP1mut
Dynein only
b
NEBD-anaphase onset timing
****
n=5
c
DHC-1::GFP signal on kinetochores of persistently laterally oriented chromosomes
n=5
DHC-1::GFP
mCh::H2b
klp-19 + ndc-80 (RNAi)
d
mCh::H2b DHC-1::GFP
PP1
KNL-1 WT
KNL-1 PP1mut
n=8
-60
-30
0
30
Time from anaphase onset (s)
klp-19 + ndc-80 + knl-1 (RNAi)
ndc-80 transgene (CHmut)
knl-1 transgene (WT or PP1mut)
e
Dynein only
+
KNL-1 WT
KNL-1 PP1mut
DHC-1::GFP kinetochore fluorescence intensity (a.u.)
Time from anaphase onset (s)

Supplementary Figure 5. Supporting data for analysis of Ndc80 module and kinetochore-localized protein phosphatase 1 perturbations.

a Interval between the Before and After K1 orientation timepoints analyzed in *Fig. 5B*. p-values are from two-tailed Student's t-tests for means: ns (not significant, $p>0.05$) (p-values 0.50 and 0.67, respectively). Error bars are the 95% CIM. *n* is the number of sister kinetochore pairs (chromosomes) analyzed. **b** Interval between NEDB and anaphase onset for indicated perturbations. This analysis provides evidence for efficacy of replacement of endogenous KNL-1 by $PP1^{mut}$ KNL-1. As shown previously[47], mitotic duration was extended, and anaphase onset delayed by ~100 s in the presence of $PP1^{mut}$ KNL-1. p-value from two-tailed Student's t-tests for means: **** ($p<0.0001$). Error bars are the 95% CIM. *n* is the number of embryos analyzed. **c** DHC-1::GFP signal on a persistently lateral chromosome 180 s after NEBD for indicated perturbation. Scale bar, 5 μm (full spindle view) and 2.5 μm (magnified region). *n* is the number of chromosomes analyzed. **d** Image sequences of persistently lateral single chromosomes and of dynein localized to its two sister kinetochores for indicated perturbations over the course of mitosis. Times are relative to anaphase onset in seconds. Scale bars, 1 μm. *n* is the number of chromosomes analyzed. **e** Quantification of the DHC-1::GFP fluorescence intensity on each kinetochore of laterally oriented chromosomes. Error bars are the 95% CIM; *n* is the number of chromosomes analyzed. Source data are provided as a Source Data file.

Supplementary Table S1: *C. elegans* Strains

STRAIN DESCRIPTION	SOURCE	IDENTIFIER
C. elegans N2 Bristol	Caenorhabditis Genetics Center	http://www.cgc.cbs.umn.edu/strain.php?id=10570
unc-119(ed3) III; ruIs32[pAZ132; pie-1/GFP::histone H2B] III; ddIs6 [GFP::tbg-1; unc-119(+)] V	Oegema et al. 2001, PMID: 11402065	TH32
unc-119(ed3) III; ltSi120[[pDC170;Pndc-80:ndc-80 reencoded; cb-unc-119(+)]II #3; ruIs32[pAZ132; pie-1/GFP::histone H2B] III; ddIs6 [GFP::tbg-1;unc-119(+)] V	Cheerambathur et al., 2013, PMID: 24231804	OD613
unc-119(ed3)III; ltSi129[pDC181;Pndc-80:NDC-80 (100,144,155AAA) reencoded; cb-unc-119(+)]II #2 ; ruIs32[pAZ132; pie-1/GFP::histone H2B] III; ddIs6 [GFP::tbg-1; unc-119(+)] V	Cheerambathur et al., 2013, PMID: 24231804	OD644
ltSi421[pDC204;Pspdl-1:spdl-1 reencoded; cb-unc-119(+)]I #1; unc-119(ed3)?III; ruIs32[pAZ132; pie-1/GFP::histone H2B] III; ddIs6 [GFP::tbg-1; unc-119(+)] V	Cheerambathur et al., 2013, PMID: 24231804	OD943
ltSi422[pDC205;Pspdl-1:spdl-1(F199A) reencoded; cb-unc-119(+)]I #1; unc-119(ed3)?III; ruIs32[pAZ132; pie-1/GFP::histone H2B] III; ddIs6 [GFP::tbg-1; unc-119(+)] V	Cheerambathur et al., 2013, PMID: 24231804	OD946
unc-119(ed3)III; ltSi560 [pPLG014; Pmex-5::GFP::his-11::tbb-2_3'UTR, tbg-1::gfp::tbb-2_3'UTR; cb-unc-119(+)]V	Kim et al., 2016, PMID: 26953348	OD1702
unc-119(ed3)III; ltSi710[pDC267;Pndc-80:NDC-80 (66,96,100,125,144,155AAAAAA) reencoded; cb-unc-119(+)]II#1 ; ruIs32[pAZ132; pie-1/GFP::histone H2B] III; ddIs6 [GFP::tbg-1; unc-119(+)] V	Cheerambathur et al., 2017, PMID: 28535376	OD2312
dhc-1(lt45; dhc-1::gfp) I; unc-119(ed3) III?; ltIs37 [pAA64; pie-1/mCherry::his-58; unc-119 (+)] IV	This Study	OD2956
lt53[knl-1::GFP::tev::loxP::3xFlag)III; ltSi711[pDC267;Pndc-80:NDC-80(66,96,100,125,144,155AAAAAA) reencoded; cb-unc-119(+)]II#1 ; ltIs37 [pAA64; pie-1/mCherry::his-58; unc-119 (+)] IV	This Study	OD3083
lt53[knl-1::GFP::tev::loxP::3xFlag)III; ltSi129[pDC181;Pndc-80:NDC-80 (100,144,155AAA) reencoded; cb-unc-119(+)]II #2; ltIs37 [pAA64; pie-1/mCherry::his-58; unc-119 (+)] IV	This Study	OD3121
klp-19(lt118[gfp::klp-19])III; ltIs3 [pAA64;pie1/mCherry::his-58; unc-119 (+)] IV	Hattersley et al., 2022, PMID: 35609608	OD3192
ndc-80(lt54[ndc-80::GFP::tev::loxP::3xFlag])IV; unc-119(ed3) III?; ltIs37 [pAA64; pie-1/mCherry::his-58; unc-119 (+)] IV	This Study	OD3300
dhc-1(lt45; dhc-1::gfp) I; ltSi120[[pDC170;Pndc-80:ndc-80 reencoded; cb-unc-119(+)]II #3; unc-119(ed3) III?; ltIs37 [pAA64; pie-1/mCherry::his-58; unc-119 (+)] IV	This Study	OD3630
dhc-1(lt45; dhc-1::gfp) I; ltSi711[pDC267;Pndc-80:NDC-80 (66,96,100,125,144,155AAAAAA) reencoded; cb-unc-119 (+)]II#1; unc-119(ed3) III?; ltIs37 [pAA64; pie-1/mCherry::his-58; unc-119 (+)] IV	This Study	OD3631
knl-1(lt53[knl-1::GFP::tev::loxP::3xFlag]))III ltSi711 [pDC267;Pndc-80:NDC-80 (66,96,100,125,144,155AAAAAA) reencoded; cb-unc-119(+)]II#1; ltIs37 [pAA64; pie-1/mCHERRY::his-58; unc-119 (+)] IV	This Study	OD3633

Supplementary Table S1: *C. elegans* Strains (continued)

STRAIN DESCRIPTION	SOURCE	IDENTIFIER
[ltSi597[pDC202 ; Pknl-1::mCherry::knl-1::knl-1 3'UTR; cb-unc-119(+)]; dhc-1 (lt45[dhc-1::gfp])]I; ltSi711[pDC267;Pndc-80:NDC-80(66,96,100,125,144,155AAAAAA) reencoded; cb-unc-119(+)]II#1; unc-119(ed3)III? ; ltIs37 [(pAA64) pie-1p::mCherry::his-58 + unc-119(+)] IV	This Study	DKC133
[dhaSi32[pDC646; Pknl-1::knl-1 reencoded (RRASA) :: mCherry::knl-13'UTR; cb-unc-119(+)]#1; dhc-1 (lt45[dhc-1 ::gfp])]I; ltSi711[pDC267;Pndc-80:NDC-80 (66,96,100,125,144,155AAAAAA reencoded; cb-unc- 119(+)]II#1; unc-119(ed3)III? ; ltIs37 [(pAA64) pie-1p ::mCherry::his-58 + unc-119(+)] IV	This Study	DKC157
ltSi711[pDC267;Pndc-80:NDC-80 (66,96,100,125,144,155AAAAAA) reencoded; cb-unc-119(+)]II#1; unc-119(ed3)III? ; ltIs37 [(pAA64) pie-1p ::mCherry::his-58 + unc-119(+)] IV; (lt39 [gfp::tev ::loxP :: 3xFlag::mdf-1])V)	This Study	DKC233
ruIs32 [pie-1p::GFP::H2B + unc-119(+)] III. ddIs6 [tbg-1::GFP + unc-119(+)] V.	This Study	DKC393
ruIs32 [pie-1p::GFP::H2B + unc-119(+)] III. ddIs6 [tbg-1::GFP + unc-119(+); he279[Δebp-1, ΔY59A8B.25, Δebp-3] V;	This Study	DKC394
rod-1(dha112 (ROD-1::mScarlet-I))I; ruIs32 [pie-1p:: GFP::H2B + unc-119(+)] III. ddIs6 [tbg-1::GFP + unc-119(+)] V.	This Study	DKC760
spdl-1(dha113 (gfp::spdl-1))II; unc-119(ed3) III; ltIs37 [pAA64; pie-1/mCherry::his-58; unc-119 (+)] IV	This Study	DKC761

Supplementary Table S2: CRISPR gRNAs

Gene No.	Name	Allele Generated	guideRNA sequence	References
Y43F4B.6	*klp-19*	*klp-19 (it118[gfp::klp-19])III*	ACCATTCATAGGCCGAGCA	Direct Integration (Waaijers et al., 2013)
C02F5.1	*knl-1*	*knl-1(lt53[knl-1::gfp::tev::loxP::3xFlag])III*	TCGAATGCTGGTGTTCTCTA	SEC (Dickinson et al., 2015)
T21E12.4	*dhc-1*	*dhc-1 (lt45[dhc-1::gfp])I*	CTACCAACGAGGAGTTGCAT	Direct Integration (Waaijers et al., 2013)
C06A8.5	*spdl-1*	*spdl-1(dha113 [gfp::spdl-1])II*	aatcagtATGCCTGACGACG	Ribonucleoprotein complex (Paix, et al., 2015)
F55G1.4	*rod-1*	*rod-1(dha112 [rod-1::mSc-I])I*	caacgaatttatTTAAGAGT	Ribonucleoprotein complex (Paix, et al., 2015)

Supplementary Table S3: dsRNAs used in this study

Gene No.	Name	Oligonucleotide (5'-3'), #1	Oligonucleotide (5'-3') #2	Template
Y43F4B.6	*klp-19*	5'- aattaaccctcactaaagg ATTGGGAGAGCTGGTGAATG-3'	5'- taatacgactcactatagg GACTTTCCTACGTGCTTCGC -3'	N2 genomic DNA
C02F5.1	*knl-1*	5'- aattaaccctcactaaagg TTCACAAACTTGGAAGCCGCTG -3'	5'- taatacgactcactatagg AATCTCGAATCACCGAAATGTC -3'	N2 genomic DNA
W01B6.9	*ndc-80*	5'- aattaaccctcactaaagg GATGACAAGTACATTCAGAGATTATACAAATGATC-3'	5'- taatacgactcactatagg GTGGTTCAAGATTCATTTGAATATTAAGTCCACTG-3'	N2 genomic DNA
F55G1.4	*rod-1*	5'- aattaaccctcactaaagg AATGCAAATCTTTTTGGATGGGAGAAAC-3'	5'- taatacgactcactatagg CATCGACGAATTTGATTCGATCAATC-3'	N2 genomic DNA
Y69A2AR.30	*mdf-2*	5'- aattaaccctcactaaagg GTGAACTGACGTCGAGAATGAG-3' -3'	5'- taatacgactcactatagg GACGGATGTAAAGACACAAAACG-3'	N2 genomic DNA
C06A8.5	*spdl-1*	5'- aattaaccctcactaaagg AACGTTACCCGAATG ACCAC-3'	5'- taatacgactcactatagg CCTAATTGAGGCATG GGTTC-3'	N2 genomic DNA
		Lowercase letters denote T3 and T7 sequences included for RNA synthesis.	Lowercase letters denote T3 and T7 sequences included for RNA synthesis.	
