# Supplementary material for: Kinetochore dynein is sufficient to biorient chromosomes and remodel the outer kinetochore
Source: Nat Commun. 2024 Oct 21;15:9085. doi: 10.1038/s41467-024-52964-5 (PMC11494143; doi:10.1038/s41467-024-52964-5)
Supplement: Supplementary file 3 — Description of additional supplementary files [file 41467_2024_52964_MOESM3_ESM.pdf]

Description of Additional Supplementary Files

Supplementary Movie 1 - Timelapse sequence of a Control one-cell embryo expressing GFP::H2b and GFP::γ-tubulin. Each movie frame is a maximum intensity projection of a 5 x 1.5 µm z-stack acquired on a spinning disk confocal microscope. Playback speed is 18X real-time.

Supplementary Movie 2 – Timelapse sequence of a “Blank Slate” one-cell embryo (<i>knl-1 + klp-19 (RNAi)</i>) expressing GFP::H2b and GFP::γ-tubulin. Each movie frame is a maximum intensity projection of a 5 x 1.5 µm z-stack acquired on a spinning disk confocal microscope. Playback speed is 18X real-time.

Supplementary Movie 3 – Timelapse sequence of a “ChrKin-only” one-cell embryo (<i>knl-1 (RNAi)</i>) expressing GFP::H2b and GFP::γ-tubulin. Each movie frame is a maximum intensity projection of a 5 x 1.5 µm z-stack acquired on a spinning disk confocal microscope. Playback speed is 18X real-time.

Supplementary Movie 4 – Timelapse sequence of a “Ndc80 module-only” embryo (<i>rod-1 + klp-19 (RNAi)</i>) expressing GFP::H2b and GFP::γ-tubulin. Each movie frame is a maximum intensity projection of a 5 x 1.5 µm z-stack acquired on a spinning disk confocal microscope. Playback speed is 18X real-time.

Supplementary Movie 5 – Timelapse sequence of a “Kinetochore Dynein module-only” embryo (<i>ndc-80 + klp-19 (RNAi); ndc-80 CHmut transgene</i>) expressing GFP::H2b and GFP::γ-tubulin. Each movie frame is a maximum intensity projection of a 5 x 1.5 µm z-stack acquired on a spinning disk confocal microscope. Playback speed is 18X real-time.

Supplementary Movie 6 - Timelapse sequence of a “Kinetochore Dynein module-only” embryo (<i>ndc-80 + klp-19 (RNAi); ndc-80 CHmut transgene</i>) expressing <i>in situ</i>-tagged DHC-1::GFP (top) and transgene-encoded mCh::H2b (bottom). The dynamics of DHC-1 on a single orienting chromosome is shown. Each movie frame is a maximum intensity projection of a 7 x 1 µm z-stack acquired on a spinning disk confocal microscope. Playback speed is 18X real-time.
