# Supplementary material for: Kinetochore dynein is sufficient to biorient chromosomes and remodel the outer kinetochore
Source: Nat Commun. 2024 Oct 21;15:9085. doi: 10.1038/s41467-024-52964-5 (PMC11494143; doi:10.1038/s41467-024-52964-5)
Supplement: Supplementary file 10 — Reporting Summary [file 41467_2024_52964_MOESM10_ESM.pdf]

nature portfolio

Corresponding author(s): Bram Prevo and Arshad Desai

Last updated by author(s): Aug 11, 2024

Reporting Summary

Nature Portfolio wishes to improve the reproducibility of the work that we publish. This form provides structure for consistency and transparency in reporting. For further information on Nature Portfolio policies, see our Editorial Policies and the Editorial Policy Checklist.

Statistics

For all statistical analyses, confirm that the following items are present in the figure legend, table legend, main text, or Methods section.

n/a	Confirmed	
☐	☒	The exact sample size (*n*) for each experimental group/condition, given as a discrete number and unit of measurement
☐	☒	A statement on whether measurements were taken from distinct samples or whether the same sample was measured repeatedly
☐	☒	The statistical test(s) used AND whether they are one- or two-sided<br>*Only common tests should be described solely by name; describe more complex techniques in the Methods section.*
☒	☐	A description of all covariates tested
☐	☒	A description of any assumptions or corrections, such as tests of normality and adjustment for multiple comparisons
☐	☒	A full description of the statistical parameters including central tendency (e.g. means) or other basic estimates (e.g. regression coefficient) AND variation (e.g. standard deviation) or associated estimates of uncertainty (e.g. confidence intervals)
☐	☒	For null hypothesis testing, the test statistic (e.g. F, t, r) with confidence intervals, effect sizes, degrees of freedom and P value noted<br>*Give P values as exact values whenever suitable.*
☒	☐	For Bayesian analysis, information on the choice of priors and Markov chain Monte Carlo settings
☒	☐	For hierarchical and complex designs, identification of the appropriate level for tests and full reporting of outcomes
☒	☐	Estimates of effect sizes (e.g. Cohen's d, Pearson's r), indicating how they were calculated

Our web collection on statistics for biologists contains articles on many of the points above.

Software and code

Policy information about availability of computer code

Data collection	Andor Revolution XD Confocal System, Andor Technology Acquisition Software. Yokogawa CSU-W1 Confocal System, Nikon Elements Acquisition Software.
Data analysis	ImageJ2 (Fiji) (version 2.14.0/1.54f) was used to extract quantitative information from image series, and processed using Excel, GraphPad Prism and / or OriginPro 2022b ((64-bit) SR1 9.9.5.171). Measurements were taken from distinct samples and assumed to be normally distributed. Statistical analysis was done using OriginPro 2022b ((64-bit) SR1 9.9.5.171) by performing two-tailed Student's t-tests for means with **** (p<0.0001) & ns (not significant, p>0.05). No statistical method was used to predetermine sample size. No data were excluded from the analyses. The experiments were not randomized. The Investigators were not blinded to allocation during experiments and outcome assessment.

For manuscripts utilizing custom algorithms or software that are central to the research but not yet described in published literature, software must be made available to editors and reviewers. We strongly encourage code deposition in a community repository (e.g. GitHub). See the Nature Portfolio guidelines for submitting code & software for further information.

Data

Policy information about availability of data

All manuscripts must include a data availability statement. This statement should provide the following information, where applicable:

- Accession codes, unique identifiers, or web links for publicly available datasets
- A description of any restrictions on data availability
- For clinical datasets or third party data, please ensure that the statement adheres to our policy

Data supporting this study are available in the primary manuscript and the supplemental material files. The C. elegans constructs and plasmids and the raw imaging data are available upon request from the lead authors. All data plotted in the graphs are provided in the Source Data file.

Research involving human participants, their data, or biological material

Policy information about studies with human participants or human data. See also policy information about sex, gender (identity/presentation), and sexual orientation and race, ethnicity and racism.

Reporting on sex and gender	N/A
Reporting on race, ethnicity, or other socially relevant groupings	N/A
Population characteristics	N/A
Recruitment	N/A
Ethics oversight	N/A

Note that full information on the approval of the study protocol must also be provided in the manuscript.

Field-specific reporting

Please select the one below that is the best fit for your research. If you are not sure, read the appropriate sections before making your selection.

☒ Life sciences ☐ Behavioural & social sciences ☐ Ecological, evolutionary & environmental sciences

For a reference copy of the document with all sections, see nature.com/documents/nr-reporting-summary-flat.pdf

Life sciences study design

All studies must disclose on these points even when the disclosure is negative.

Sample size	Sample sizes were chosen according to well-accepted experimental standards in the field.
Data exclusions	No data was excluded.
Replication	All attempts at replication were successful.
Randomization	No drug treatment involved. Strains are selected based on relevant genotype. Animal selected from NGM plate at random. One-cell embryo selected from dissected individual at random.
Blinding	Blinding was not relevant for this study. Strains are selected based on relevant genotype.

Reporting for specific materials, systems and methods

We require information from authors about some types of materials, experimental systems and methods used in many studies. Here, indicate whether each material, system or method listed is relevant to your study. If you are not sure if a list item applies to your research, read the appropriate section before selecting a response.

Materials & experimental systems

n/a	Involved in the study
☒	☐ Antibodies
☒	☐ Eukaryotic cell lines
☒	☐ Palaeontology and archaeology
☐	☒ Animals and other organisms
☒	☐ Clinical data
☒	☐ Dual use research of concern
☒	☐ Plants

Methods

n/a	Involved in the study
☒	☐ ChIP-seq
☒	☐ Flow cytometry
☒	☐ MRI-based neuroimaging

Animals and other research organisms

Policy information about studies involving animals; ARRIVE guidelines recommended for reporting animal research, and Sex and Gender in Research

Laboratory animals	C. elegans adult nematodes
Wild animals	Study did not involve wild animals.
Reporting on sex	N/A. Only C. elegans hermaphrodites were involved.
Field-collected samples	Study did not involve samples collected from the field.
Ethics oversight	No ethical approval or guidance is required for work with C. elegans.

Note that full information on the approval of the study protocol must also be provided in the manuscript.

Plants

Seed stocks	N/A
Novel plant genotypes	N/A
Authentication	N/A
